# Functional Role of N-Terminal Extension of Human AP Endonuclease 1 In Coordination of Base Excision DNA Repair via Protein–Protein Interactions

**DOI:** 10.3390/ijms21093122

**Published:** 2020-04-28

**Authors:** Nina Moor, Inna Vasil’eva, Olga Lavrik

**Affiliations:** 1 Institute of Chemical Biology and Fundamental Medicine, Siberian Branch of the Russian Academy of Sciences, 630090 Novosibirsk, Russia; moor@niboch.nsc.ru (N.M.); iva@niboch.nsc.ru (I.V.); 2 Novosibirsk State University, 630090 Novosibirsk, Russia

**Keywords:** APE1, protein–protein interactions, base excision repair, multifunctional disordered protein, fluorescence techniques

## Abstract

Human apurinic/apyrimidinic endonuclease 1 (APE1) has multiple functions in base excision DNA repair (BER) and other cellular processes. Its eukaryote-specific N-terminal extension plays diverse regulatory roles in interaction with different partners. Here, we explored its involvement in interaction with canonical BER proteins. Using fluorescence based-techniques, we compared binding affinities of the full-length and N-terminally truncated forms of APE1 (APE1NΔ35 and APE1NΔ61) for functionally and structurally different DNA polymerase β (Polβ), X-ray repair cross-complementing protein 1 (XRCC1), and poly(adenosine diphosphate (ADP)-ribose) polymerase 1 (PARP1), in the absence and presence of model DNA intermediates. Influence of the N-terminal truncation on binding the AP site-containing DNA was additionally explored. These data suggest that the interaction domain for proteins is basically formed by the conserved catalytic core of APE1. The N-terminal extension being capable of dynamically interacting with the protein and DNA partners is mostly responsible for DNA-dependent modulation of protein–protein interactions. Polβ, XRCC1, and PARP1 were shown to more efficiently regulate the endonuclease activity of the full-length protein than that of APE1NΔ61, further suggesting contribution of the N-terminal extension to BER coordination. Our results advance the understanding of functional roles of eukaryote-specific protein extensions in highly coordinated BER processes.

## 1. Introduction

Apurinic/apyrimidinic endonuclease 1 (APE1) is an essential protein in mammals with multiple functions in base excision DNA repair (BER), regulation of gene expression, RNA metabolism, and other specific cellular processes [1,2,3]. BER is the primary mechanism for correcting apurinic/apyrimidinic (AP) sites (created through N-glycosidic bond cleavage), modified bases, and single-strand breaks (SSBs) [4,5]. The major enzymatic function of APE1 in BER is the incision of AP sites, one of the most abundant types of oxidative DNA damage [6]. Additional activities of APE1, 3′-diesterase, and 3′-5′-exonuclease contribute to removing terminal blocking groups in BER DNA intermediates and to proofreading DNA mismatches introduced by DNA polymerase β (Polβ) [1,3]. APE1 is also capable of incising DNA at certain base lesions (nucleotide incision repair) and RNA at abasic site and specific regions, as well as of processing abasic and oxidized ribonucleotides embedded in the DNA [1,2,3,7,8]. A redox activity of APE1 is responsible for the regulation of DNA-binding activities of different transcription factors [1,2]. The DNA repair and redox activities of APE1 can play a collaborative role in different processes [9,10]. Interactions with multiple protein partners that modulate diverse enzymatic activities of APE1 and mediate its regulatory function in transcription are highly interconnected and dynamically regulated through various post-translational modifications of APE1 [1,2,11,12,13]. Dysregulation of multifunctional activities of APE1 is associated with various human pathologies, making APE1 a potential therapeutic target [1,5,12,14].

**Figure 1:**
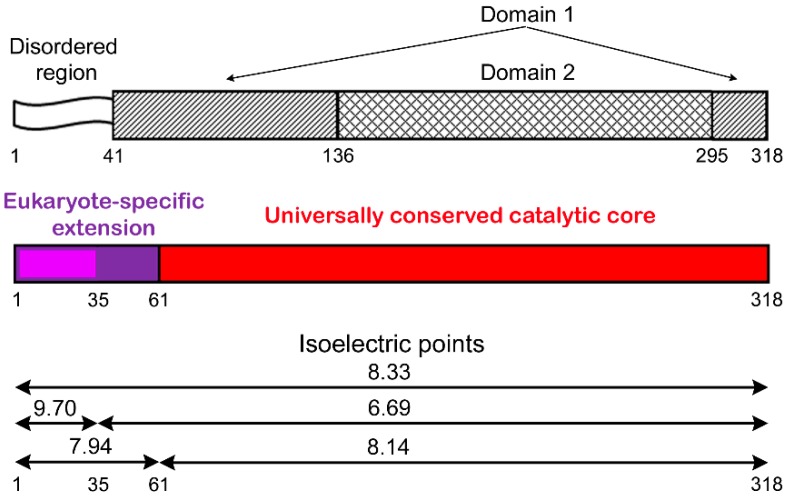
Structural organization of human apurinic/apyrimidinic endonuclease 1 (APE1). Protein is composed of two structural domains and disordered N-terminal region invisible in crystal structures [15]. Sequence 62–318 responsible for AP endonuclease activity is conserved in pro- and eukaryotes; first 35 residues of N-terminal eukaryote-specific extension (residues 1–61) are highly conserved in mammals [16]. Isoelectric points (calculated using ExPASy proteomics server) of full-length protein, its N-terminally truncated forms (APE1NΔ35, APE1NΔ61), the entire extension, and its mammalian-specific fragment are presented.

The human APE1 is composed of two structural domains and a disordered N-terminal region [15], formed mostly by the sequence highly conserved in mammals (Figure 1). The catalytically active core responsible for the enzyme activities in BER is created by the universally conserved sequence 62–318 [16]. The region required for the redox activity of APE1 is in the N-terminal domain [1]. The unique disordered region comprises the nuclear localization signal and multiple post-translational modification sites. This region, enriched in lysine residues, contributes to APE1 interaction with various DNA/RNA structures and to acetylation-mediated modulation of the enzyme DNA repair activity in vitro and in vivo [17,18,19,20,21]. Ubiquitination and acetylation of specific Lys residues of the N-terminal region modulate the expression level and different functions of APE1 in vivo via proteolytic degradation or limited N-terminal proteolysis, respectively [22,23]. The conserved catalytic core and the N-terminal extension are both required for the APE1 function at telomeric DNA substrates and their protective protein complexes [24,25]. The N-terminal extension of APE1 contributes to a different extent to the interaction with proteins involved in RNA processing and ribosome biogenesis [26]; its interaction with nucleophosmin (NPM1) is very unstable to be detected in the absence of the remaining C-terminal portion [20]. The first 35 residues are critical for the physical interaction of APE1 with X-ray repair cross-complementing protein 1 (XRCC1) functioning as a scaffold protein of BER [27]. However, involvement of the N-terminal extension of APE1 in the interaction with other canonical BER proteins still remains unexplored.

We demonstrated the usefulness of fluorescence-based techniques for the detection and quantification of physical interactions between various BER proteins, and for the detection of modulation of protein–protein interactions by DNA intermediates [28]. In the present study, we used the same approaches to explore the role of the N-terminal extension of APE1 in coordination of mammalian BER process via protein–protein interactions. The relative binding affinities of the full-length and two N-terminally truncated forms of APE1 (APE1NΔ35 and APE1NΔ61) for three functionally and structurally different BER proteins, downstream enzyme Polβ, scaffold XRCC1 protein, and poly(ADP-ribose) polymerase 1 (PARP1) responsible for detecting SSBs and initiating their repair, were determined in the absence and presence of model DNA intermediates of BER, and the DNA-induced rearrangements of the protein–protein complexes were examined. Additionally, the influence of the N-terminal truncation on the functional cooperation between APE1 and Polβ in the absence and presence of accessory XRCC1 and PARP1 proteins was explored.

## 2. Results

### 2.1. Contribution of N-terminal Extension of Human APE1 to the Interaction with Canonical BER Proteins

**Figure 2:**
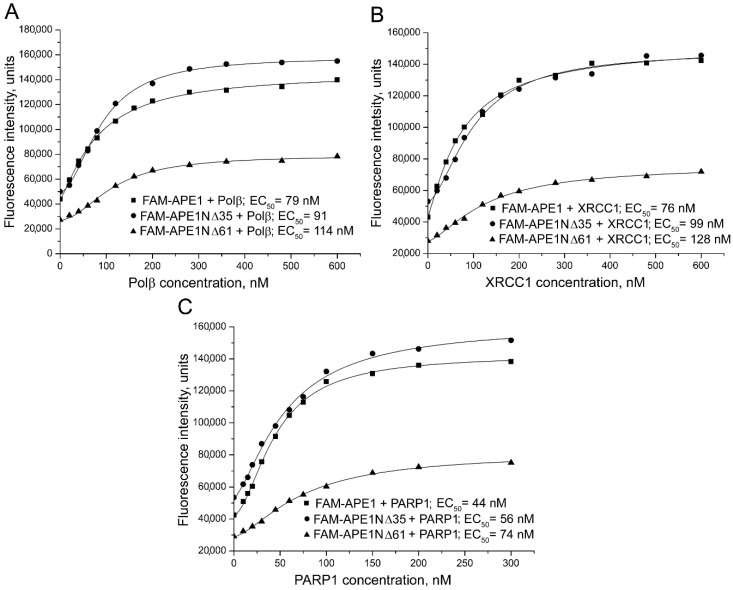
Fluorescence titration of 5(6)-carboxyfluorescein (FAM)-labelled APE1, APE1NΔ35, and APE1NΔ61 with (**A**) DNA polymerase β (Polβ) (**B**) X-ray repair cross-complementing (XRCC1) protein 1, and (**C**) poly(ADP-ribose) polymerase 1 (PARP1). FAM-labelled protein (40 nM) excited at 482 nm in absence or presence of increasing concentrations of protein partner, and relative fluorescence intensities monitored at 530 nm. Curves show best fits (R^2^ values met or exceeded 0.98) of four-parameter equation; EC_50_ values derived from respective curves are presented. Data shown are representative of at least three independent experiments.

To explore involvement of the N-terminal extension of human APE1 in the interaction with other BER proteins, and to quantify the contribution of the entire eukaryote-specific region and its disordered mammalian-conserved fragment to the binding affinities, fluorescence-titration experiments were performed using fluorescein-labelled full-length APE1 and its N-terminally truncated forms (APE1NΔ35 and APE1NΔ61). The proteins N-terminally labelled with 5(6)-carboxyfluorescein (FAM) were prepared as previously described [28] and are detailed in Supplementary Materials. The change in the fluorescence intensity of a FAM-labelled protein (FAM-APE1, FAM-APE1NΔ35, or FAM-APE1NΔ61) was monitored in the presence of unlabeled protein partners added at increasing concentrations (Figure 2).

The fluorescence intensity of the FAM-labelled APE1 and its truncated forms increased in the presence of Polβ, XRCC1, or PARP1, indicating that the local environment of the fluorophore changed upon protein–protein association. Apparent equilibrium dissociation constants of the complexes determined by nonlinear regression analyses as effective concentrations (EC_50_ values) of the protein partners at the half-maximal increase in fluorescence intensity [29] are presented in Table 1. Removal of the first 35 amino acid residues slightly decreased the affinity of APE1 for all three proteins (a 1.2–1.3-fold increase in EC_50_). Removal of the entire eukaryote-specific extension (residues 1–61) produced more significant effects: The binding affinity of APE1 for Polβ decreased 1.4-fold, and the affinity for PARP1 and XRCC1 decreased 1.7-fold.

**Figure 3:**
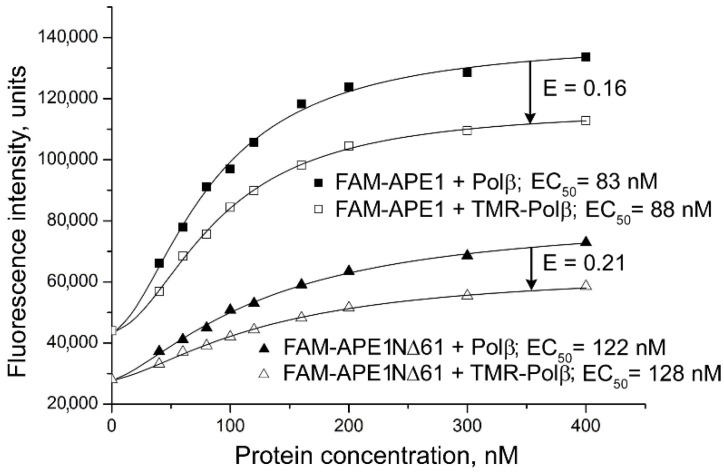
Characterization of Polβ interaction with APE1 and its truncated form by FRET. The FAM-labelled protein (40 nM FAM-APE1 or FAM-APE1NΔ61) excited at 482 nm in the absence or presence of increasing concentrations of unlabeled or TMR-labelled Polβ. FRET efficiency calculated from the fractional decrease (shown by arrow) of fluorescence intensity of the donor due to the presence of acceptor E = 1 – F_da_/F_d_, where F_da_ and F_d_ are fluorescence intensities measured in the presence of identical subsaturating concentrations of TMR-Polβ or Polβ (open or filled symbols, respectively), and determined at saturation by fitting to four-parameter equation. EC_50_ and E values determined for respective complexes are shown.

To further explore influence of the N-terminal truncation on physical contacts between APE1 and BER proteins, we performed fluorescence resonance energy transfer (FRET) experiments. FRET is characterized by the efficiency of energy transfer (ET) from a fluorescent donor to an acceptor, which depends on the extent of the spectral overlap between donor emission and acceptor absorption, on the distance between the two fluorophores and on their relative orientation [30]. We chose fluorescein and tetramethylrhodamine as the donor–acceptor pair previously used to characterize interactions between BER proteins [28]. FAM-labelled APE1, APE1NΔ35, or APE1NΔ61 was titrated with Polβ, unlabeled or labelled with 5(6)-carboxytetramethylrhodamine (TMR) (Figure 3). The fluorescence intensity of the FAM-labelled protein increased less in the presence of TMR-Polβ than in the presence of Polβ, indicating participation of the donor- and acceptor-labelled proteins in FRET. FRET efficiencies determined for the FAM-APE1NΔ35‒TMR-Polβ and FAM-APE1‒TMR-Polβ pairs were identical (Table 1). FRET efficiency determined for the FAM-APE1NΔ61‒TMR-Polβ pair was appreciably (6%) higher, indicating that, in this complex, the distance between two fluorophore probes and/or their relative orientation are more favorable for FRET. Analogous FRET measurements were performed for the other complexes. FRET signals detected for the XRCC1 complexes with the truncated forms of APE1 were lower than for the respective complex with the full-length APE1 (Table 1). On the other hand, efficiencies of FRET for the TMR-PARP1 pairs with FAM-APE1N35 and FAM-APE1NΔ61 were higher than for the respective pair with FAM-APE1. The differences in detected FRET signals evidently reflected changes in the localization of the FAM-labelled N-terminus within complexes formed by the full-length and N-terminally truncated forms of APE1.

Taken together, these results indicate that the N-terminal extension of human APE1 is involved in the interactions of APE1 with other BER proteins. Small effects produced by the N-terminal truncation on the binding affinity constants suggest that the interaction domain for proteins is basically formed by the conserved catalytic core of APE1, and the N-terminal extension mediates dynamic interactions. 

### 2.2. Involvement of N-Terminal Extension of APE in DNA-Dependent Modulation of Protein–Protein Interactions

Using fluorescence-based approaches, we showed in a previous study that DNA intermediates of BER induce rearrangements of various protein–protein complexes and modulate the strength of the interaction [28]. To explore the possible involvement of the N-terminal region of APE1 in this modulation, fluorescence titration and FRET experiments with the full-length and truncated forms of APE1 were performed in the absence and presence of model DNA ligands (shown in Figure S1). A double-stranded DNA with a synthetic abasic site (a tetrahydrofuran residue, F), AP-DNA, is an initial BER substrate of APE1. A 1-nucleotide gapped DNA with a 5′-F group at the margin of the gap models a product of the APE1-catalyzed incision (AP-DNA inc) and a stable analog of the Polβ substrate not processed by the 5′-deoxyribose phosphate lyase activity. A 1-nucleotide-gapped DNA (gap-DNA) and AP-DNA inc are substrates of both APE1 (in the 3′-5′-exonuclease reaction) and Polβ (in the DNA repair synthesis). APE1 and Polβ bind with the highest affinity AP-DNA and gap-DNA, respectively; their affinities for AP-DNA inc are relatively high and comparable, and those for the nonsubstrate double-stranded DNA are very low [31]. XRCC1 prefers 1-nt-gapped and nicked oligonucleotide duplexes, while there is no significant difference in the strength of PARP1 interaction with different duplex models of damaged DNA due to preferential binding to blunt ends [32,33,34,35,36]. Functional interactions between APE1, Polβ, XRCC1, and PARP1 on various DNA intermediates, contributing to regulation of BER, were demonstrated in previous studies [27,31,37,38,39,40,41,42] and are further detailed in Section 2.3. 

**Figure 4:**
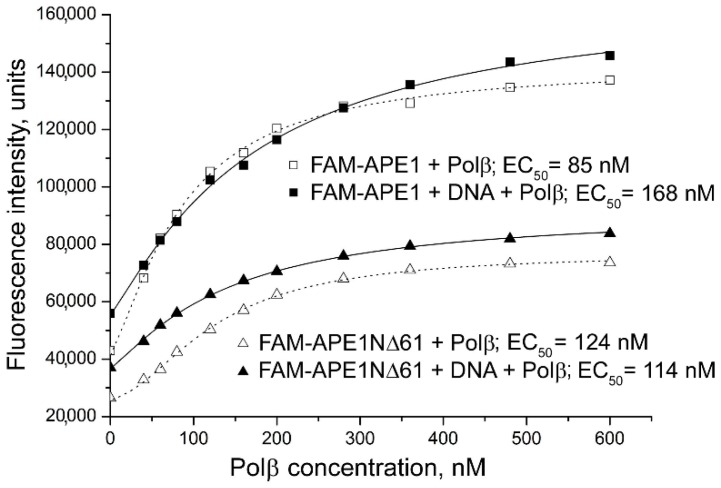
Influence of DNA intermediate of base excision DNA repair (BER) on the interaction of APE1 and its truncated form with Polβ. Fluorescence titration of FAM-labelled APE1 or APE1NΔ61 (40 nM) with Polβ was performed in the absence (open symbols) or presence (filled symbols) of incised AP-DNA (400 nM); higher values of F_0_ measured in DNA presence are indicative of protein–DNA binding. Curves show best fits of four-parameter equation with R^2^ values exceeding 0.98; EC_50_ values derived from respective curves are presented.

Binding of the truncated forms of APE1 with each protein partner in the absence and presence of a given DNA intermediate was explored in parallel with the full-length APE1 (Figure 4). From these experiments, the effects produced by the model DNA on the quantitative characteristics of protein–protein interactions, apparent binding affinity constant and FRET efficiency, were determined (Table 2). AP-DNA inc produced the highest effect on the binding affinity of FAM-APE1 for Polβ (two-fold decrease), while its effects detected for the respective complexes formed by FAM-APE1NΔ35 and FAM-APE1NΔ61 were not statistically significant. The presence of intact AP-DNA revealed no significant effect on the binding affinity of both the full-length and truncated forms of APE1 for Polβ. Gap-DNA produced a small effect (1.3-fold decrease detected as statistically significant) for the FAM-APE1 complex. Changes in the efficiency of FRET detected for complexes of the truncated forms of APE1 with TMR-Polβ in the presence of the various DNA intermediates (a 7–9% decrease) were indicative of the DNA-induced rearrangement of the complexes. The different DNAs produced significantly different changes in the FRET signals only in the case of the full-length protein complex (a 5% increase vs. a 6–11% decrease). These combined results suggest that conformational changes in the APE1–Polβ complex caused by DNA binding involve both the conserved catalytic core of APE1 and the N-terminal extension, with the last being important to control the strength of interaction between proteins in the complex with damaged DNA depending on its type.

Both the structure and stability of the FAM-APE1 complex with XRCC1 were detected as described previously to be modulated in the presence of gap-DNA [28]: The binding affinity and FRET efficiency increased 1.5-fold and by 8%, respectively (Table 2). However, the parameters of FAM-APE1 interaction with XRCC1 were not affected by the presence of intact AP-DNA. Here, we revealed that interaction of FAM-APE1NΔ35 and FAM-APE1NΔ61 with XRCC1 was appreciably influenced by both gap-DNA and AP-DNA. For each of the truncated forms, the effects produced by the different DNAs on the strength of interaction with XRCC1 and on the FRET signals were very similar. Clearly, integrity of the N-terminal portion of APE1 is important to control the mode of APE1–XRCC1 interaction during the processing of distinct DNA intermediates.

We compared the influence of AP-DNA and gap-DNA on the interaction of the full-length and truncated forms of APE1 with PARP1 (Table 2). Significant effects on the interaction of FAM-APE1 with PARP1 were detected only in the presence of AP-DNA: Binding affinity decreased 1.7 fold and FRET efficiency increased by 5% (Table 2). Both AP-DNA and gap-DNA produced similar positive effects (a 1.4-fold increase) on the strength of interaction between FAM-APE1NΔ61 and PARP1. In the case of FAM-APE1NΔ35, no statistically significant effects of DNA intermediates on the protein interaction with PARP1 were detected. Interestingly, EC_50_ values determined for the complexes of FAM-APE1 and FAM-APE1NΔ61 with PARP1 were identical in the presence of gap-DNA, but the affinity of FAM-APE1 for PARP1 in the presence of AP-DNA was practically identical with that of FAM-APE1NΔ61 in the absence of DNA (76 nM vs. 75 nM). Evidently, the N-terminal extension is responsible for the destabilizing effect of the preferred binding substrate of APE1 on APE1–PARP1 interaction.

### 2.3. Influence of N-Terminal Truncation of APE1 on Functional Cooperation between BER Proteins

**Figure 5:**
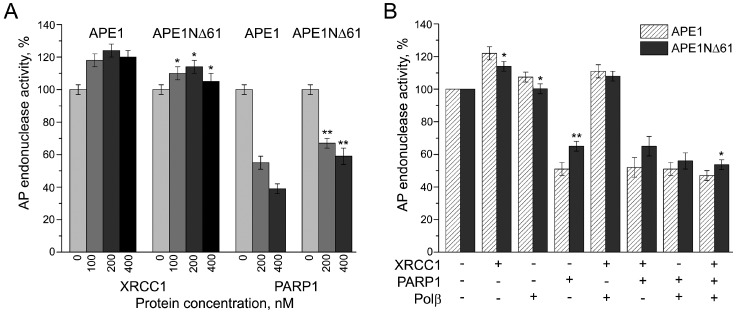
Comparison of effects produced by Polβ, XRCC1, and PARP1 on the AP endonuclease activity of APE1 and APE1NΔ61. Activity in incision of AP-DNA was measured in the absence or presence of Polβ, XRCC1, and PARP1, added separately at varied concentrations (indicated on the X axis, (**A**) or in different combinations at a constant concentration of each (200 nM) (**B**). In each set of experiments, activities were determined as initial rates of AP-DNA incision normalized to that of APE1/APE1NΔ61 in the absence of other proteins (taken as 100%). One representative experiment of the activity measurements is shown in Figure S2. Data are the mean (± SD) of three independent measurements. Effects detected for APE1NΔ61, which were statistically different from those for APE1, are marked *p* < 0.05 (*), *p* < 0.01 (**).

Numerous studies showed that the enzymatic activities of APE1 on various DNA intermediates of BER are modulated by protein partners: The AP-endonuclease activity by Polβ and PARP1, and the 3*′*-5*′*-exonuclease activity by Polβ, PARP1, and XRCC1 [31,38,39,40,41,42]. The nucleotidyltransferase activity of Polβ is stimulated by APE1 and XRCC1, and inhibited by PARP1 [37,38,39,41,42]. In light of our results described above, we explored the possible involvement of the N-terminal extension of APE1 in the functional coupling of BER proteins to each other. First, we examined the influence of Polβ, XRCC1, and PARP1 on the AP endonuclease activity of APE1NΔ61 in comparison with the full-length enzyme (Figure 5). The activity of APE1 and APE1NΔ61 was enhanced by XRCC1 and inhibited by PARP1 in a concentration-dependent manner (Figure 5A). The effects observed for the full-length APE1 exceeded those for the truncated form at all tested concentrations of XRCC1 and PARP1. Stimulation of the AP endonuclease activity by Polβ shown previously by others [39] was detectable only for the full-length APE1 (Figure 5B). The stimulating effects produced by XRCC1 and Polβ present separately or together were fully suppressed by the addition of PARP1. The differences in effects detected for APE1 and APE1NΔ61 in the presence of all binary combinations of the protein partners were statistically insignificant. At the same time, the inhibiting effect on the activity of APE1 detected in the presence of the ternary protein combination was higher than that for APE1NΔ61. Thus, the N-terminal extension of APE1 being not essential for the major catalytic activity of APE1 may potentially contribute to the regulation of this activity by the BER protein partners.

**Figure 6:**
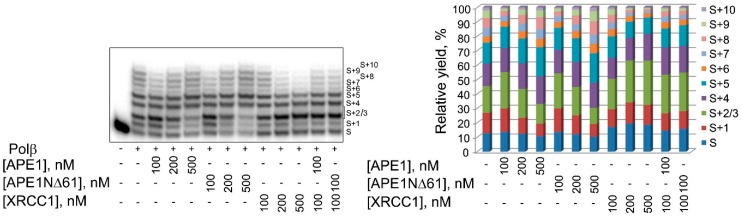
Comparison of effects produced by APE1 and APE1NΔ61 on Polβ activity. Polβ activity in DNA synthesis was measured on gap-DNA in the absence or presence of APE1, APE1NΔ61, and XRCC1, added separately at varied concentrations (100–500 nM) or together at a constant concentration (100 nM APE1/APE1NΔ61 and 100 nM XRCC1). Histograms present relative amounts of substrate (S) and products of gap-filling (S+1) and strand-displacement synthesis (S+2 to S+10). Reaction was performed as described in Materials and Methods. Data are representative of three independent experiments with very similar results.

We next compared APE1 and APE1NΔ61 in modulating the nucleotidyltransferase activity of Polβ, using gap-DNA as the substrate (Figure 6). The concentration-dependent effects on the yields of products of gap-filling and strand-displacement DNA synthesis produced by the full-length and truncated forms of APE1 were very similar. No appreciable difference between the two APE1 forms was detected when their influence on the Polβ-catalyzed DNA synthesis in the presence of XRCC1, a strong binding partner of Polβ [28], was compared. Data suggested that the N-terminal extension of APE1 is not essential for the stimulation of Polβ-catalyzed DNA repair synthesis.

### 2.4. Contribution of N-Terminal Extension of APE1 to AP-DNA Binding

**Figure 7:**
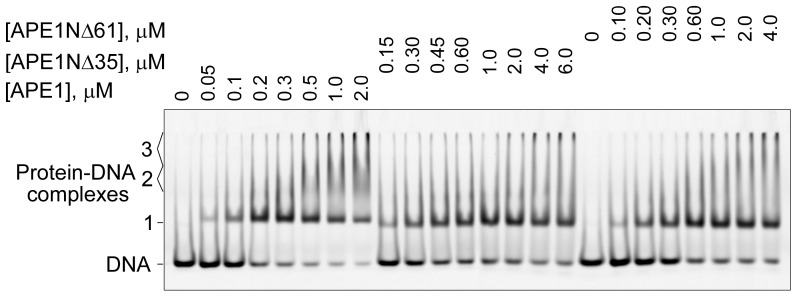
Comparison of APE1 and its N-terminally truncated forms in binding AP site-containing DNA. FAM-labelled AP-DNA was incubated with increasing concentrations of APE1/APE1NΔ35/APE1NΔ61 in the absence of metal ions. After incubation, protein–DNA complexes were separated from free DNA by native gel electrophoresis as described in Materials and Methods. Data are representative of three independent experiments.

Participation of the N-terminal extension of human APE1 in binding the intact and incised AP site-containing DNA was demonstrated by chemical footprinting assay [17]. Recently, oligomerization of APE1 on undamaged DNA and its dependence on the presence of the N-terminal extension was revealed by electron microscopy analysis [43]. However, there are no quantitative data on the relative contribution of the N-terminal extension and the conserved catalytic core to the protein affinity for the AP-DNA substrate. Here, we compared the full-length and truncated forms of APE1 in their modes and strength of AP-DNA binding using electrophoretic-mobility-shift assay (EMSA). Three types of complexes with different electrophoretic mobilities were detected for APE1 (Figure 7), while only two of them, designated as Complexes 1 and 3, could be visualized for APE1NΔ35 and APE1NΔ61. Fast migrating Complex 1 obviously represents a monomeric protein–DNA complex that is stabilized by interactions between AP-DNA and the conserved catalytic portion of APE1. Slow migrating Complex 3 most likely results from oligomerization of the monomeric complex via protein–protein interactions as previously proposed [43]. Complex 2 with intermediate mobility, formed exclusively by the full-length protein, is obviously stabilized by interactions between DNA and the N-terminal extension of APE1; the positively charged N-terminus may additionally neutralize the negative charge of DNA, thereby decreasing complex mobility. Another possibility is the dimerization of APE1 on the DNA, which is highly unstable in the absence of the N-terminus to be detected by the nonequilibrium EMSA technique. The affinity of APE1 for DNA estimated from the protein-concentration dependence of the total amount of bound DNA is 1.8-fold higher as compared to those of APE1NΔ35 and APE1NΔ61 (Table 3). At concentrations of APE1/APE1NΔ35/APE1NΔ61 around the EC_50_ value, the only detectable complex was Complex 1, indicating a lower stability of Complexes 2 and 3. Oligomerization was visualized at lower concentrations of APE1 as compared to those of APE1NΔ35 and APE1NΔ61. Our data provide quantitative evidence that the N-terminal extension of APE1 contributes to the additional stabilization of the complex with AP-DNA, and may control the mode of protein–DNA association (mono- and/or oligomerization) depending on protein concentration.

## 3. Discussion

BER is an exceptionally efficient process evolved by mammalian cells to correct the most abundant DNA lesions. The BER process can proceed along one of different subpathways that involve distinct enzymes and accessory proteins/cofactors (Figure S3). The efficient repair of damaged DNA via the multistep process of each of the subpathways requires the coordinated action of enzymes catalyzing the sequential individual reactions [2,3,4]. Coordination is facilitated by multiple protein–protein interactions, reviewed previously [13,44]. Physical interaction and functional interplay between two major BER enzymes, APE1 and Polβ, were shown by various approaches [13,31,38,41,45]. XRCC1 functioning as a nonenzymatic scaffold protein directly interacts with multiple enzymatic components of BER, using all structural domains and flexible linkers [13,46]. PARP1, responsible for assembling DNA repair complexes via automodification at sites of DNA damage, forms direct and PAR-mediated contacts with various BER proteins, and modulates the catalytic activities of BER enzymes [4,13,35,40,41,47,48]. Physical and functional interaction between APE1 and PARP1 detected by various in vitro techniques as well as ADP-ribosylation of APE1 catalyzed by PARP1 via the unusual mechanism controlled by the structure of damaged DNA provide evidence of functional assistance between the proteins during DNA repair [28,41,45,49,50,51,52,53].

Here, we explored the contribution of the N-terminal extension of human APE1 to the interaction with Polβ, XRCC1, and PARP1, using fluorescence-based quantitative techniques. The eukaryote-specific extension of APE1 (residues 1–61) is dispensable for endonuclease activity [16], but essential for DNA binding and acetylation-mediated modulation of the enzyme DNA repair activity in vitro and in vivo [17,18,19,21]. Our data, obtained under true equilibrium conditions, clearly showed that the removal of the entire N-terminal extension (APE1NΔ61) or its mammalian-conserved fragment (APE1NΔ35) decreased the affinity of APE1 for all three proteins to small but measurable extents (Table 1). The small effects produced by N-terminal truncation (less than two-fold) indicate that the C-terminal catalytic core of human APE1, highly conserved through evolution, mainly contributes to the interaction with proteins, and the N-terminal extension forms an additional low-affinity binding site. Our results contradict previous data that demonstrated that the first 35 residues are absolutely required for APE1–XRCC1 interaction assayed by far-Western blotting [27]. This discrepancy could have resulted from disadvantages of the far-Western blotting assay: A significantly lower isoelectric point of APE1NΔ35 (6.69 vs. 8.33 for full-length APE1, Figure 1) might reduce efficiency of protein electrotransfer to nitrocellulose membrane [54]. The considerable part of the eukaryote-specific extension of APE1 is disordered and invisible in the crystal structure of the full-length protein [55]. Numerous studies showed that disordered protein regions are involved in dynamic interactions, contributing little to overall binding energy, but being essential for regulatory functions [56,57].

Disordered terminal tails characterized by clustering of positively charged residues are present predominantly in mammalian DNA-binding proteins [56]. They were proposed to have similar functions in the initial scanning of DNA by BER proteins via transient electrostatic binding, which is followed by specific binding of the lesion in the active site [56]. The N-terminus of human APE1 was shown to stabilize interaction with nonspecific RNA/DNA structures [18,20] and to increase the extent of APE1 oligomerization on undamaged long DNA [43]. Its involvement in binding a specific AP site-containing DNA substrate was demonstrated by protein footprinting with a lysine-reactive probe [16]. In our EMSA binding experiments, we detected a metastable, unique to the full-length APE1, complex with AP-DNA, which accumulated along with the most stable complex formed independently of N-terminal truncation (Figure 7). Compared to APE1, the truncated APE1NΔ35 and APE1NΔ61 forms were shown to form less stable monomeric and oligomeric complexes. These results provide the first quantitative evidence that the N-terminal extension forms an additional low-affinity DNA binding site in the whole protein and contributes to the stabilization of various forms of the APE1–DNA complex.

Previously, we demonstrated that the strength of APE1 interaction with Polβ, XRCC1, and PARP1, and the structure of APE1–protein complexes are modulated by model BER DNA intermediates to different extents depending on the type of damaged DNA [28]. This finding motivated us to explore the involvement of eukaryote-specific extension in this modulation, particularly in light of its capacity to dynamically bind both the DNA and the protein partners. The distinct effects produced by various DNA intermediates on protein–protein affinity binding constants of the full-length APE1 turned out to not be distinguishable for the N-terminally truncated forms (Table 2). These results indicate that the N-terminal extension of human APE1 plays a primary role in the DNA-dependent modulation of the strength of APE1 interaction with both the downstream enzyme and the accessory proteins of BER. This role is obviously related to the coordination of enzymatic functions during BER that is governed by DNA-binding specificity and protein–protein interactions. The interaction between APE1 and Polβ is stronger in the presence of intact AP-DNA than in the complex mimicking a step after the APE1-catalyzed incision, suggesting that the incised DNA intermediate is more effectively passed to Polβ immediately during the incision step. The conformationally flexible N-terminus of APE1, being highly adaptable to both the protein and DNA structure, may differently modulate direct and DNA-mediated protein–protein interactions in the ternary complex with the specific DNA depending on the relative affinities of the protein partners for the DNA. Indeed, changes in FRET signals reflecting structural DNA-induced rearrangements of the APE1–Polβ complex are quite dissimilar for different DNA intermediates. Footprinting experiments showed that intact and incised AP-DNAs protect the N-terminus of APE1 in accordance with the different stabilities of the complexes, but their protective action was undetectable in the ternary complexes with Polβ [17], suggesting higher flexibility of the N-terminal extension in the APE1–DNA–Polβ complexes than in the respective APE1–DNA complexes. The product release is the rate-limiting step of the APE1-catalyzed AP site incision, and this limitation can be overcome by the stimulatory action of Polβ [39]. Involvement of Polβ and PARP1 in the dynamics of APE1 function in vivo was recently shown [58]. Here, we showed that the major function of APE1 is also activated by XRCC1 (Figure 5), previously detected to stimulate the 3′-diesterase activities of APE1 [27]. A comparison of the effects produced by Polβ, XRCC1, and PARP1 on the activity of APE1 and APE1NΔ61 revealed more efficient regulation by all proteins (present separately) for the full-length enzyme. These results provide further evidence that the N-terminal extension of APE1 is involved in interactions with canonical BER proteins promoting the coordination of the process.

APE1 enhances the 5’-deoxyribose phosphate(dRp)-lyase and nucleotidyltransferase activities of Polβ [31,41]. Our comparison of APE1 and APE1NΔ61 in modulating the catalytic activity of Polβ in DNA synthesis, tested on the canonical substrate of short-patch BER, revealed no dependence of the stimulatory function of APE1 on the integrity of its N-terminal portion (Figure 6). No appreciable difference between the two APE1 forms was detected when their effects were compared in the presence of XRCC1, capable of forming the ternary complex with these enzymes [28]. The question of whether the N-terminal extension of APE1 contributes to the functional coupling with Polβ upon the processing of other DNA intermediates, more efficient substrates of APE1 in the 3′-5′-exonuclease reaction [59], remains open for future research.

APE1 stimulates the catalytic activities of different DNA glycosylases, upstream BER enzymes, via accelerating product release [43,60,61,62,63,64]. The coordination mechanism via protein–protein interactions was proposed for TDG (thymine DNA glycosylase) and MYH (MutY homolog DNA glycosylase) [60,62]; binding sites for MYH do not involve the N-terminal extension of APE1 [62]. Other studies suggested no physical interaction between APE1 and DNA glycosylases. The importance of the N-terminal extension of APE1 for the stimulatory function is explained by its participation in protein oligomerization along the DNA, proposed to promote dissociation of DNA glycosylases from the complex with product [43,64]. Recently we detected and characterized new, never previously predicted [65,66], activity of APE1 in binding poly(ADP-ribose) (PAR) [53]. Both the N-terminal extension and the conserved catalytic core of APE1 were found to be involved in PAR binding, with the first contributing for the most part to the interaction with small linear polymers. We propose that the interaction of APE1 with PAR, found to be less efficient as compared to that of XRCC1, may contribute to assembling DNA repair complexes during the PARP1-dependent processing of SSBs. Present and previous studies provide evidence that the N-terminal extension of APE1 may perform multiple, diverse functions in the coordination of BER.

## 4. Materials and Methods

### 4.1. Protein Expression and Purification

Recombinant human APE1, its N-terminally truncated forms (APE1NΔ35 and APE1NΔ61), and rat Polβ were produced by expression in *Escherichia coli* BL21(DE3)pLysS (BL21 E. coli strain carrying the lambda DE3 lysogen and the pLysS plasmid, which expresses T7 lysozyme) and purified as described previously [67,68,69]. Human PARP1 and human protein XRCC1 were produced by expression in *E. coli* Rosetta (DE3) and purified as described [70,71]. APE1 and Polβ expression vectors were kindly provided by S.H. Wilson (National Institute of Health, North Carolina, USA). The plasmid constructs used to express APE1NΔ35 and APE1NΔ61 were kindly provided by A.A. Ishchenko (Groupe Réparation de l’ADN, UMR 8126 CNRS, Univ Paris-Sud, Institut Gustave Roussy, France). The XRCC1 expression vector was a generous gift from J.P. Radicella (UMR217 CNRS/CEA, France). The plasmid construct used to express PARP1 was kindly provided by M. Satoh (Laval University, Quebec, Canada). The purified proteins were dialyzed against a solution containing 50 mM Tris-HCl, pH 8.0, 100 mM NaCl, 5 mM DTT (dithiotheitol), and 40% glycerol, and stored at −30 °C.

### 4.2. Fluorescence Studies of Protein–Protein Interactions

Fluorescent labelling of proteins on the terminal amino group using succinimidyl esters of 5(6)-carboxyfluorescein (FAM-SE) and 5(6)-carboxytetramethylrhodamine (TMR-SE) was performed as described [28]. The stoichiometry of protein labelling did not exceed 1 mole of dye per mole of protein, and FAM/TMR-labelled enzymes retained their specific activities (as detailed in Supplementary Materials, Table S1). Binding of proteins to each other was examined by fluorescence titration experiments. Fluorescence intensities of solutions of the FAM-labelled protein (APE1, APE1NΔ35, or APE1NΔ61) at a fixed concentration were measured in the absence and presence of varied concentrations of the partner (Polβ/PARP1/XRCC1), in a binding buffer containing 50 mM HEPES (4-(2-hydroxyethyl)-1-piperazineethanesulfonic acid), pH 8.0, 100 mM NaCl, and 4 mM DTT. Fluorescence intensity of the samples was measured in Corning 384-well black polypropylene assay plates using a CLARIOstar multifunctional microplate reader (BMG LABTECH GmbH, Germany); fluorescence probes were excited at 482 nm (482-16 filter), and the relative fluorescence intensities were detected at the emission maximum (530 nm, 530-40 filter). All measurements were carried out in duplicate for each specific condition, and the average values of fluorescence (with mean deviations not exceeding 2%) were taken for data analysis performed using MARS (Multivariate Adaptive Regression Splines) Data Analysis Software (BMG LABTECH GmbH, Germany). Data were plotted (F vs. C) and fitted by four-parameter logistic equation

F = F_0_ + (F_∞_ – F_0_)/[1 + (EC_50_/C)^n^] (1)

where F is the fluorescence intensity of a solution containing the FAM-labelled protein and the binding partner at a given concentration (C), F_0_ is the fluorescence of a solution of the labelled protein alone, F_∞_ is the fluorescence of the labelled protein saturated with the partner, EC_50_ is the concentration of the binding partner at which F – F_0_ = (F_∞_ – F_0_)/2, and n is the Hill coefficient denoting the slope of the nonlinear curve.

To detect protein–protein interactions by the FRET approach, the fluorescence intensity of the FAM-labelled protein (donor probe) was measured in the absence and presence of varied concentrations of the TMR-labelled protein (acceptor probe). Measurements were performed in two series: (1) Donor probe + unlabeled partner, and (2) donor probe + acceptor probe (FRET pair). FRET efficiency (E) was calculated from the fractional decrease of the donor fluorescence (F_d_) due to the presence of the acceptor (F_da_)

E = 1 – F_da_/F_d_. (2)

In protein–protein binding experiments performed in the presence of model DNAs (prepared as described in Supplementary Materials), the FAM-labelled protein (40 nM) was premixed with the desired DNA, and fluorescence intensity was taken as a starting F_0_ value. Conditions for the formation of ternary complexes were optimized by varying DNA concentration from 120 to 600 nM. Titration experiments were performed in binding buffer supplemented with 10 mM EDTA (ethylenediaminetetraacetic acid) (to suppress the AP-endonuclease and the 3′-5′-exonuclease activities of APE1 and its truncated forms).

### 4.3. AP Endonuclease Activity Assay

The endonuclease activity of APE1 and APE1NΔ61 was assayed in reaction mixtures (25–50 μL) containing 50 mM Tris-HCl, pH 8.0, 50 mM NaCl, 10 mM MgCl_2_, 1 mM DTT, 0.1 mg/mL BSA (bovine serum albumin), and 100 nM 5’-^32^P-labelled AP-DNA substrate (32 base-pair oligonucleotide with a synthetic AP site, prepared as described in Supplementary Materials). The reaction mixtures were preassembled on ice; when indicated, they were supplemented with Polβ, XRCC1, and PARP1 (at concentrations specified in the figure legends). Reaction was initiated by adding APE1 (APE1NΔ61) to a final concentration of 0.5 nM; the reaction mixtures were incubated at 37 °C for 0.5–4 min and terminated by the addition of denaturing PAGE sample buffer and heating for 2 min at 90 °C. The reaction products were separated by electrophoresis in 20% denaturing polyacrylamide gels. The gels were imaged on a Typhoon FLA 9500, and the amounts of DNA substrate and product were quantified using Quantity One Basic software.

### 4.4. DNA Synthesis by Polβ

The activity of Polβ in DNA synthesis was assayed in reaction mixtures (10 μL) containing 50 mM Tris-HCl, pH 8.0, 50 mM NaCl, 6 mM MgCl_2_, 50 nM 5’-^32^P-labelled gap-DNA substrate (32 base-pair oligonucleotide with a one-nucleotide gap prepared as described in Supplementary Materials), and 50 nM Polβ. Reaction mixtures were preassembled on ice; when indicated, they were supplemented with APE1, APE1NΔ61, and XRCC1 (at concentrations specified in the figure legends). The reaction was initiated by adding a mixture of dATP (2’-deoxyadenosine 5’-triphosphate), dGTP (2’-deoxyguanosine 5’-triphosphate), dTTP (2’-deoxythymidine 5’-triphosphate) and dCTP (2’-deoxycytidine 5’-triphosphate) to a final concentration of 10 μM for each nucleotide. Reaction mixtures were incubated at 37 °C for 30 min, and terminated by the addition of denaturing PAGE sample buffer and heating for 2 min at 90 °C. The reaction products were separated by electrophoresis in 20% denaturing polyacrylamide gels. The gels were imaged on a Typhoon FLA 9500, and the amounts of DNA substrate and products were quantified using Quantity One Basic software.

### 4.5. Electrophoretic-Mobility-Shift Assay

The affinity of APE1, APE1NΔ35, and APE1NΔ61 for AP-DNA was measured by electrophoretic-mobility-shift assay (EMSA). A desired protein (added at increasing from 0.05 to 2–6 µM concentrations) was incubated with FAM-labelled AP-DNA (50 nM) in a 10 μL mixture containing 50 mM Tris-HCl, pH 8.0, 100 mM NaCl, and 1 mM DTT at 4 °C for 30 min. After the addition of glycerol and bromophenol blue (to a final concentration of 5% and 0.1%, respectively), the incubation mixtures were electrophoresed at 4 °C on 5% nondenaturing PAG (polyacrylamide gel) in a 30 mM Tris-Borate-EDTA buffer. Gels were imaged on a Typhoon FLA 9500. Free and complexed DNA bands were quantified using Quantity One Basic software. To subtract free DNA co-migrated with the protein-bound DNA (due to partial overlapping of their migration zones as a result of DNA smearing) the portion of free DNA in the protein-containing samples (determined from radioactivity distribution between migration zones assigned to the free DNA and its complexes with proteins) was normalized to the portion of free DNA in the control sample without the protein. Data were fitted to a Hill equation: θ = θ_∞_/[1 + (EC_50_/C)^n^], where θ is the portion of bound DNA (calculated as the complex amount divided by total DNA amount) at a given concentration (C) of the protein, θ_∞_ is the maximal extent of DNA binding (i.e., the portion of DNA bound at saturating protein concentration), EC_50_ is the protein concentration at which θ = θ_∞_/2, and n is the Hill coefficient.

## Figures and Tables

**Table 1:** Binding parameters of protein–protein interactions determined for full-length and truncated forms of APE1 by fluorescence-based approaches.

Labelled Protein ^a^	Protein Partner	EC_50_^b^, nM	Effect on Affinity ^c^	E ^d^
FAM-APE1	Polβ	84 ± 7		0.16 ± 0.02
FAM-APE1NΔ35	Polβ	97 ± 8 *	1.2	0.16 ± 0.02
FAM-APE1NΔ61	Polβ	120 ± 11 **	1.4	0.22 ± 0.02 *
FAM-APE1	XRCC1	76 ± 8		0.14 ± 0.01
FAM-APE1NΔ35	XRCC1	100 ± 10 **	1.3	0.11 ± 0.01 *
FAM-APE1NΔ61	XRCC1	130 ± 12 ***	1.7	0.09 ± 0.01 **
FAM-APE1	PARP1	45 ± 5		0.04 ± 0.01
FAM-APE1NΔ35	PARP1	57 ± 5 **	1.3	0.13 ± 0.02 **
FAM-APE1NΔ61	PARP1	75 ± 7 ***	1.7	0.10 ± 0.01 **

^a^ Titration experiments were performed at the constant 40 nM concentration of FAM-labelled protein. Underlined data for FAM-APE1 (previously published [28]) reproduced to compare binding parameters of full-length and truncated forms. ^b^ Parameters derived from titration curves by fitting to four-parameter equation, where EC_50_ is half-maximal effective concentration of the protein partner, at which F – F_0_ = (F_∞_ – F_0_)/2, where F, the fluorescence intensity of a solution containing the FAM-labelled protein and the binding partner at a given concentration (C); F_0,_ the fluorescence of a solution of the labelled protein alone; F_∞_, the fluorescence of the labelled protein saturated with the binding partner. Values are the mean (± SD) of at least three independent experiments. Values determined for complexes formed by truncated forms of APE1 with each protein partner statistically different from those of the full-length protein: *p* < 0.05 (*), *p* < 0.01 (**), *p* < 0.001 (***); *t*-test, *n* = 3–4. ^c^ Effect of N-terminal truncation of APE1 on protein–protein affinity determined as the ratio of EC_50_ values for complexes formed by the truncated APE1 and by the full-length APE1 with each binding partner. ^d^ Fluorescence resonance energy transfer (FRET) efficiency calculated from the fractional decrease of fluorescence intensity, E = 1 – F_da_/F_d_, where F_da_ and F_d_ are fluorescence intensities of donor-labelled protein measured in the presence of the acceptor-labelled or the unlabeled protein partner, respectively. Values are the mean (± SD) of three independent experiments. Values determined for truncated forms of APE1, which were statistically different from those of the full-length protein in the respective complexes, are marked *p* < 0.05 (*), *p* < 0.01 (**).

**Table 2:** Effects of BER DNA intermediates on protein–protein interactions.

Labelled Protein ^a^	DNA ^a^	Protein Partner	EC_50_^b^, nM	Effect on Affinity ^c^	Effect on FRET Efficiency ^d^
FAM-APE1	AP-DNA	Polβ	92 ± 7	1.1	+0.05 *
FAM-APE1	AP-DNA inc	Polβ	170 ± 13 ***	2.0	–0.06 **
FAM-APE1	gap-DNA	Polβ	110 ± 8 *	1.3	–0.11 **
FAM-APE1NΔ35	AP-DNA	Polβ	91 ± 7	0.94	–0.08 **
FAM-APE1NΔ35	AP-DNA inc	Polβ	110 ± 10	1.1	–0.0 8**
FAM-APE1NΔ61	AP-DNA	Polβ	110 ± 10	0.92	–0.08 **
FAM-APE1NΔ61	AP-DNA inc	Polβ	130 ± 11	1.1	–0.09 **
FAM-APE1NΔ61	gap-DNA	Polβ	140 ± 13	1.2	–0.07 *
FAM-APE1	AP-DNA	XRCC1	78 ± 7	1.0	–0.01
FAM-APE1	gap-DNA	XRCC1	51 ± 4 **	0.67	+0.08 **
FAM-APE1NΔ35	AP-DNA	XRCC1	68 ± 6 **	0.68	–0.03 *
FAM-APE1NΔ35	gap-DNA	XRCC1	65 ± 6 **	0.65	–0.03 *
FAM-APE1NΔ61	AP-DNA	XRCC1	99 ± 8 *	0.76	–0.06 **
FAM-APE1NΔ61	gap-DNA	XRCC1	97 ± 8 *	0.75	–0.05 **
FAM-APE1	AP-DNA	PARP1	76 ± 6 **	1.7	+0.05 **
FAM-APE1	gap-DNA	PARP1	54 ± 4	1.2	+0.02
FAM-APE1NΔ35	AP-DNA	PARP1	49 ± 4	0.86	–0.03
FAM-APE1NΔ35	gap-DNA	PARP1	48 ± 4	0.84	+0.03
FAM-APE1NΔ61	AP-DNA	PARP1	55 ± 5 *	0.73	–0.02
FAM-APE1NΔ61	gap-DNA	PARP1	54 ± 4 *	0.72	+0.04 *

^a^ Titration experiments performed at the constant concentrations of FAM-labelled protein (40 nM) and DNA (400 nM). Underlined data for FAM-APE1 (previously published [28]) reproduced to compare binding parameters of full-length and truncated forms. ^b^ Parameters derived from titration curves by fitting to four-parameter equation. Values are the mean (± SD) of at least three independent experiments. Values determined for each protein pair in the presence of DNA, which were statistically different from the respective value in the absence of DNA, are marked *p* < 0.05 (*), *p* < 0.01 (**), *p* < 0.001 (***). ^c^ Effect of DNA on protein–protein affinity determined as the ratio of EC_50_ values in the presence or absence of DNA. ^d^ Increase (+) or decrease (–) in FRET efficiency between FAM- and TMR (5(6)-carboxytetramethylrhodamine)-labelled proteins in the presence of DNA. Statistically significant changes in E values produced by DNA are marked *p* < 0.05 (*), *p* < 0.01 (**).

**Table 3:** Parameters of AP-DNA binding by full-length and truncated forms of APE1.

Protein	EC_50_^a^, nM	Maximal Extent of Binding ^a^
APE1	0.12 ± 0.02	0.96 ± 0.03
APE1NΔ35	0.20 ± 0.03	0.77 ± 0.03
APE1NΔ61	0.21 ± 0.03	0.84 ± 0.03

^a^ Parameters derived from electrophoretic-mobility-shift-assay (EMSA) data as described in Materials and Methods; EC_50_ is the effective protein concentration, at which the extent of DNA binding is half of the maximal extent. Values are the mean (± SD) of at least three independent experiments.

## Supplementary Materials

Supplementary materials can be found at https://www.mdpi.com/1422-0067/21/9/3122/s1. Figure S1. Structures of DNA ligands used in the study; Figure S2. Influence of Polβ, XRCC1, and PARP1 on the AP endonuclease activity of APE1 and APE1NΔ61. Figure S3. Scheme illustrating stages of BER subpathways.

## Abbreviations

APE1	apurinic/apyrimidinic endonuclease 1
BER	base excision repair
AP site	apurinic/apyrimidinic site
SSB	single-strand break
Polβ	DNA polymerase β
XRCC1	X-ray repair cross-complementing protein 1
PARP1	poly(ADP-ribose) polymerase 1
APE1NΔ35/APE1NΔ61	APE1 without 35/61 N-terminal amino acid residues
FAM	5(6)-carboxyfluorescein
TMR	5(6)-carboxytetramethylrhodamine
FRET	Fluorescence resonance energy transfer
AP-DNA/gap-DNA	DNA with a synthetic AP site/1-nucleotide gap
FAM-SE/TMR-SE	succinimidyl ester of 5(6)-carboxyfluorescein/5(6)-carboxytetramethylrhodamine
