# Supplementary material for: Functional Role of N-Terminal Extension of Human AP Endonuclease 1 In Coordination of Base Excision DNA Repair via Protein–Protein Interactions
Source: Int J Mol Sci. 2020 Apr 28;21(9):3122. doi: 10.3390/ijms21093122 (PMC7247576; doi:10.3390/ijms21093122)
Supplement: Supplementary file 1 [file ijms-21-03122-s001.pdf]

International Journal of *Molecular Sciences*

MDPI

Supplementary Materials

Functional role of N-terminal extension of human AP endonuclease 1 in coordination of base excision DNA repair via protein–protein interactions

Nina Moor [1], Inna Vasil'eva [1] and Olga Lavrik [1,2,*]

[1] Institute of Chemical Biology and Fundamental Medicine, Siberian Branch of the Russian Academy of Sciences, Novosibirsk, 630090, Russia; moor@niboch.nsc.ru (N.M.); iva@niboch@nsc.ru (I.V.); lavrik@niboch.nsc.ru (O.L.)

[2] Novosibirsk State University, Novosibirsk, 630090, Russia

* Correspondence: lavrik@niboch.nsc.ru

1. Supplementary Materials and Methods

1.1. Synthesis and characterization of fluorescently labelled proteins

Functionally active FAM-labelled APE1, TMR-labelled Polβ, XRCC1, and PARP1 were prepared in reaction conditions optimized in our previous study [1]. To prepare functionally active FAM-labelled APE1NΔ35 and APE1NΔ61, the reaction conditions found for APE1 were additionally verified by varying the molar excess of reagent over the protein, and the incubation time. Molar excess of the reactive probe over the protein in the range of 1.6–2-fold is optimal to prepare fully active FAM-APE1NΔ35 and FAM-APE1NΔ61 with the labelling stoichiometry of ~0.7–0.9 mole of dye per 1 mole of protein (Table S1). The extent of protein labelling was quantified by determining dye and protein amounts in the sample. The dye concentration was measured spectrophotometrically using the absorption coefficients of $68x10^3$ $M^{-1}cm^{-1}$ at 494 nm for 5(6)-FAM and $65x10^3$ $M^{-1}cm^{-1}$ at 555 nm for 5(6)-TMR [2]. The protein concentration was determined using the Bradford assay [3].

Table S1. Labelling of APE1, APE1NΔ35, APE1NΔ61, Polβ, PARP1, and XRCC1.

Protein	Reagent	Molar ratio reagent:protein; incubation time [a]	Stoichiometry of labelling, mole of dye/mole of protein [b]	Activity, % [b,c]
APE1	FAM-SE	1.6:1; 13 h	0.86 ± 0.02	98 ± 2
APE1NΔ35	FAM-SE	1.6:1; 13 h	0.94 ± 0.03	98 ± 2
APE1NΔ61	FAM-SE	2:1; 13 h	0.68 ± 0.02	90 ± 2
Polβ	TMR-SE	1.6:1; 13 h	0.79 ± 0.02	101± 4
XRCC1	TMR-SE	3:1; 13 h	0.84 ± 0.03	
PARP1	TMR-SE	2:1; 13 h	0.79 ± 0.03	97 ± 3

[a] The reaction mixture contained 100 mM MES, pH 7.0, 150 mM NaCl, 100 μM protein (with exception of XRCC1 labelled at 50 μM) and varied concentrations of the reactive probe. FAM-SE, TMR-SE – N-succinimidyl ester of 5(6)-carboxyfluorescein or of 5(6)-carboxytetramethylrhodamine.

[b] Values are the mean (± SD) of measurements of three independent preparations.

[c] The catalytic activity of the dye-labelled enzyme is normalized to the activity of the respective unlabeled enzyme, measured in identical conditions as described in the text.

The AP endonuclease activity of FAM-labelled APE1, APE1NΔ35, and APE1NΔ61 in comparison with their unlabeled counterparts was tested as described in the main text (Materials and Methods). The enzymatic activity of TMR-labelled Polβ in comparison with the unlabeled enzyme was verified on activated DNA. The reaction mixture contained 50 mM Tris-HCl, pH 8.0, 50 mM NaCl, 10 mM $MgCl_2$, 5 mM DTT, 10 μM dNTP (dATP, dGTP, dTTP), 2 μM [α-^{32}P]dCTP, and 2 A_{260}/ml of activated DNA (high-molecular mass DNA treated with DNAse I). The reaction was initiated by adding enzyme to a final concentration of 250 nM. The reaction mixtures were incubated at 37 °C for 1–10 min. The amount of ^{32}P-labelled DNA in aliquots was determined by trichloroacetic acid precipitation and subsequent counting. The enzymatic activity of TMR-PARP1 in comparison with the unlabeled PARP1 was verified in autopoly(ADP-ribosyl)ation. The standard reaction mixture contained 50 mM Tris-HCl, pH 8.0, 100 mM NaCl, 10 mM $MgCl_2$, 5 mM DTT, 5 μM [^{32}P]NAD^+, and 40 nM gap-DNA. The reaction was initiated by adding enzyme to a final concentration of 40 nM. The reaction mixtures, after incubation at 37 °C for 2–10 min, were terminated by the addition of SDS-PAGE sample buffer and heating for 2 min at 95 °C. The reaction products were analyzed by 10% SDS-PAGE with subsequent Phosphor imaging.

1.2. Preparation of DNA ligands for enzyme activity assays and binding experiments

DNA oligonucleotides were synthesized and purified in the Laboratory of Medicinal Chemistry, Institute of Chemical Biology and Fundamental Medicine, Novosibirsk, Russia. Double-stranded 32-mer oligonucleotides (Figure S1) containing a one-nucleotide gap (gap-DNA) or a synthetic intact (AP-DNA) or incised AP site (AP-DNA inc) were prepared by annealing the complementary chain (or the respective upstream and downstream primers) to the template oligonucleotide mixed in equimolar ratios. The mixture was heated at 90 °C for 5 min, and then slowly cooled down to room temperature. The amount of duplex DNA was controlled by native 10% polyacrylamide gel electrophoresis. Radioactively and fluorescently labelled AP-DNA and gap-DNA for enzymatic activity assays and binding experiments were prepared using 5'-^{32}P-labelled or 5'-FAM-labelled F-containing oligonucleotide for AP-DNA and 5'-^{32}P-labelled upstream primer for gap-DNA.

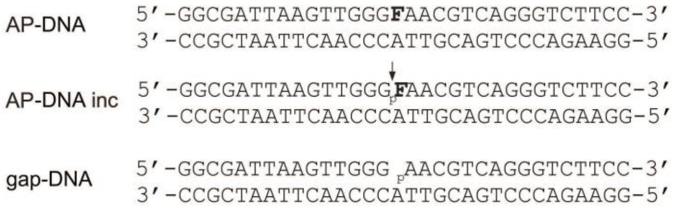

Figure S1. Structures of DNA ligands used in the study. F is 3-hydroxy-2-hydroxymethyltetrahydrofuran residue; a single covalent discontinuity in one stand is shown by arrow.

2. Supplementary Figures for Results and Discussion

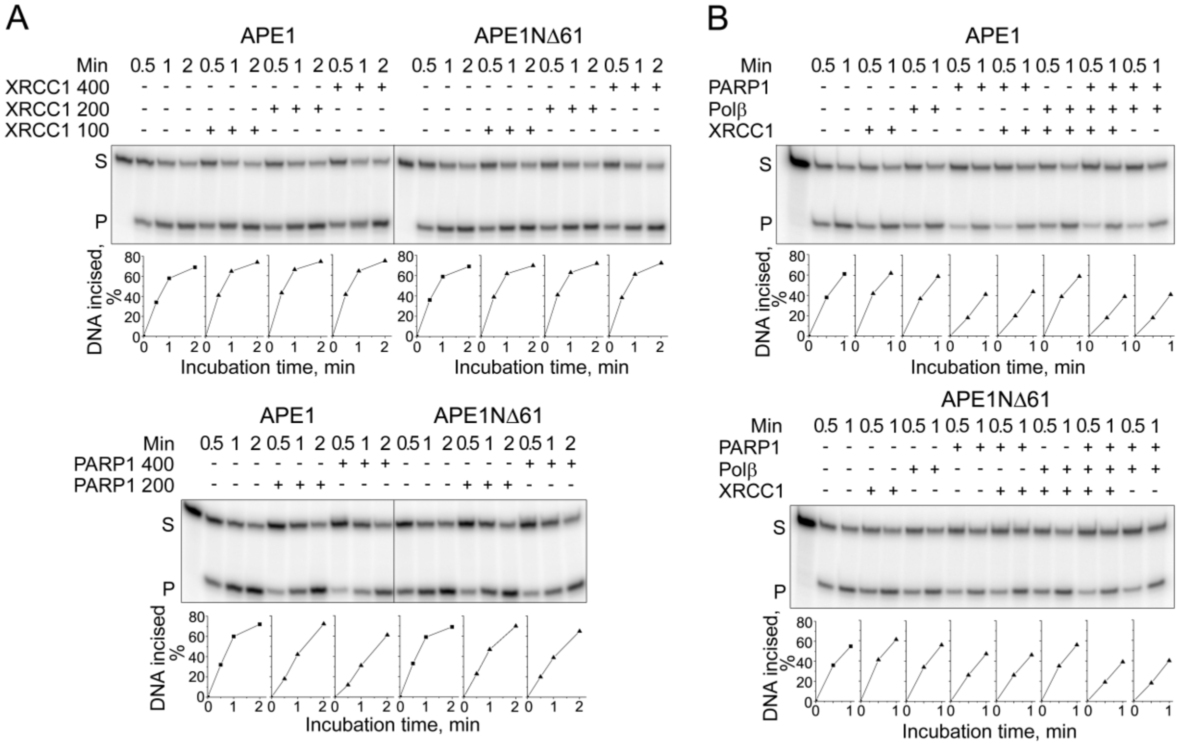

Figure S2. Influence of Polβ, XRCC1, and PARP1 on the AP endonuclease activity of APE1 and APE1NΔ61. 5'-^{32}P-labelled AP-DNA was incubated with 0.5 nM APE1 or APE1NΔ61 for the indicated time period (min), in the absence (-) or presence (+) of Polβ, XRCC1, and PARP1 at indicated varied concentrations (100–400 nM, panel **A**) or at the constant concentration of each protein (200 nM, panel **B**). The other reaction conditions, separation of substrate and product, and quantification are described in the main text (Materials and Methods). Graphs below gel images show time courses of the reaction. Data are representative of at least three independent experiments.

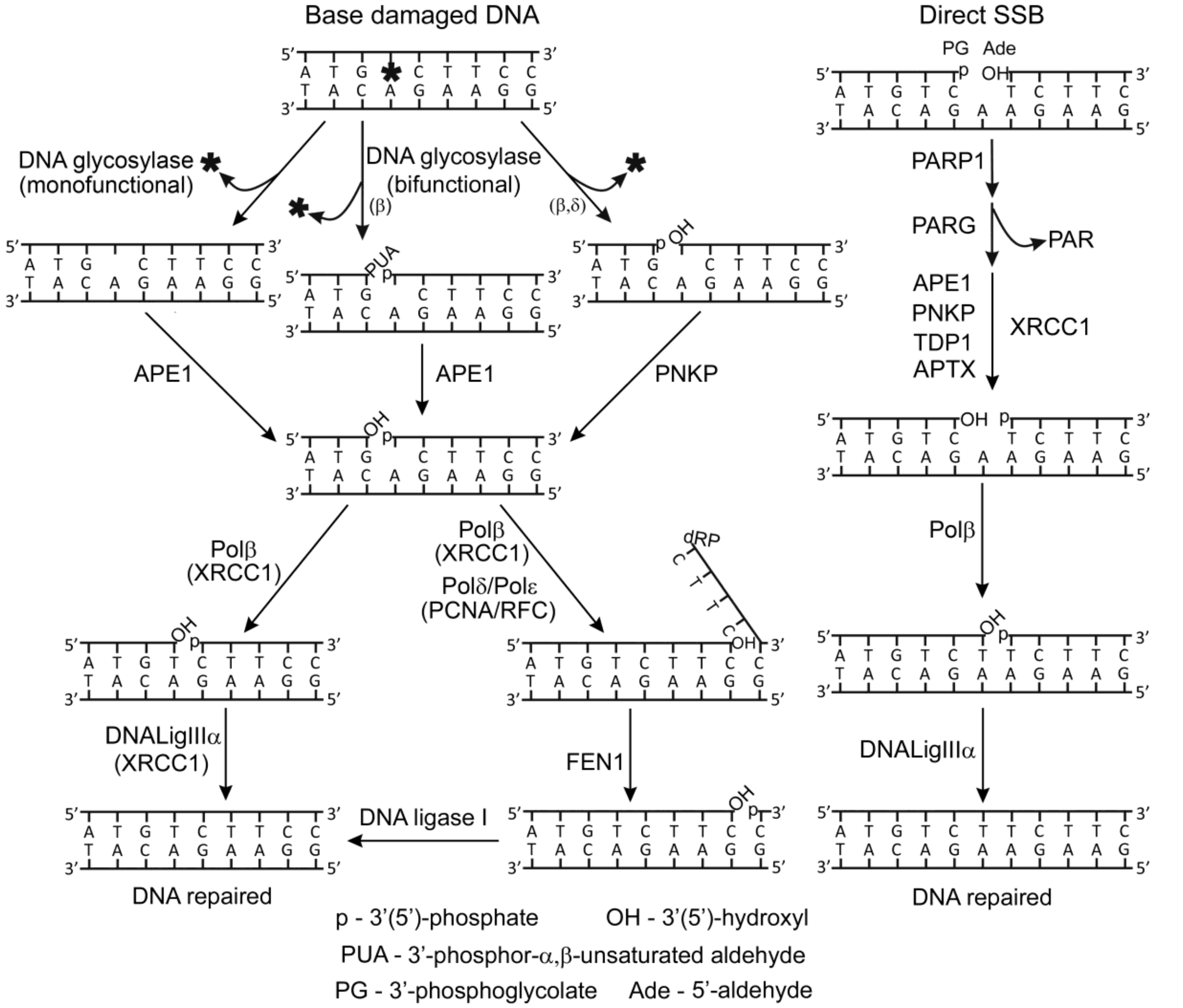

Figure S3. Scheme illustrating stages of BER subpathways. Repair of base-damaged DNA is initiated by the activity of a DNA glycosylase producing an AP site or a single-strand break (SSB) with a damaged terminus, repaired subsequently by APE1 or polynucleotide kinase 3'-phosphatase (PNKP). Further gap filling and final DNA ligation are processed through short-patch or long-patch repair involving Polβ and DNA ligase IIIα (DNALigIIIα) in complex with XRCC1 or Polβ/Polδ/Polε, flap endonuclease 1 (FEN1), and DNA ligase I respectively. Direct SSBs (arising by sugar damage or during activity of topoisomerase 1) are detected by PARP1; PARP1 binding and activation promotes processing of SSB by downstream repair factors.

Supplementary References

1. Moor, N.A., Vasil'eva, I.A., Anarbaev, R.O., Antson, A.A., Lavrik, O.I. Quantitative characterization of protein-protein complexes involved in base excision DNA repair. *Nucleic Acids Res*. **2015**, *43*, 6009–6022.
2. Haugland, R.P. *The Handbook – a Guide to Fluorescent Probes and Labeling Technologies*, 10th ed; Molecular Probes, Eugene: Oregon, USA, 2005.
3. Bradford, M.M. A rapid and sensitive method for the quantitation of microgram quantities of protein utilizing the principle of protein-dye binding. *Anal. Biochem*. **1976**, *72*, 248–254.
